# Retinol from hepatic stellate cells via STRA6 induces lipogenesis on hepatocytes during fibrosis

**DOI:** 10.1186/s13578-020-00509-w

**Published:** 2021-01-06

**Authors:** Injoo Hwang, Eun Ju Lee, Hyomin Park, Dodam Moon, Hyo-Soo Kim

**Affiliations:** 1 Molecular Medicine & Biopharmaceutical Sciences, Graduate School of Convergence Science and Technology, and College of Medicine, Seoul National University, Seoul, Republic of Korea; 2 Biomedical Research Institute, Seoul National University Hospital, 101 DeaHak-ro, JongRo-gu, Seoul, 03080 Republic of Korea; 3 Department of Internal Medicine, Seoul National University College of Medicine, 101 DeaHak-ro, JongRo-gu, Seoul, 03080 Republic of Korea

**Keywords:** Retinol, Hepatocytes, Stimulated by retinoic acid 6 (STRA6), Lipogenesis, Liver fibrosis

## Abstract

**Background:**

Hepatic stellate cells (HSCs) are activated in response to liver injury with TIF1γ-suppression, leading to liver fibrosis. Here, we examined the mechanism how reduction of TIF1γ in HSCs induces damage on hepatocytes and liver fibrosis.

**Method:**

Lrat:Cas9-ERT2:sgTif1γ mice were treated Tamoxifen (TMX) or wild-type mice were treated Thioacetamide (TAA). HSCs were isolated from mice liver and analyzed role of Tif1γ. HepG2 were treated retinol with/without siRNA for Stimulated by retinoic acid 6 (STRA6) or Retinoic acid receptor(RAR)-antagonist, and LX2 were treated siTIF1γ and/or siSTRA6. TAA treated mice were used for evaluation of siSTRA6 effect in liver fibrosis.

**Results:**

When we blocked the Tif1γ in HSCs using Lrat:Cas9-ERT2:sgTif1γ mice, retinol is distributed into hepatocytes. Retinol influx was confirmed using HepG2, and the increased intracellular retinol led to the upregulation of lipogenesis-related-genes and triglyceride. This effect was inhibited by a RAR-antagonist or knock-down of STRA6. In the LX2, TIF1γ-suppression resulted in upregulation of STRA6 and retinol release, which was inhibited by STRA6 knock-down. The role of STRA6-mediated retinol transfer from HSCs to hepatocytes in liver fibrosis was demonstrated by in vivo experiments where blocking of STRA6 reduced fibrosis.

**Conclusions:**

Retinol from HSCs via STRA6 in response to injury with TIF1γ-reduction is taken up by hepatocytes via STRA6, leading to fat-deposition and damage, and liver fibrosis. 
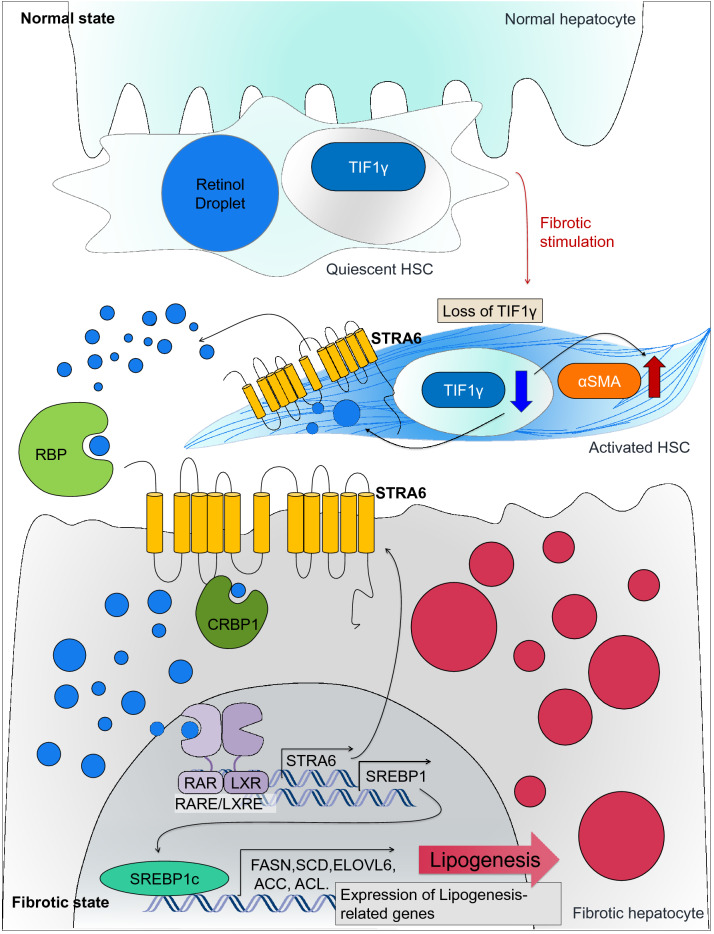

## Background

Liver cirrhosis results in severe symptoms as a result of a complex process involving fibrosis [1, 2]. The drugs market for liver cirrhosis is expected to rise gradually to an estimated value of approximately 66 billion US dollars by 2026, registering a compound annual growth rate of 10.5% in the 2019–2026 forecast period (https://www.databridgemarketresearch.com/reports/global-liver-cirrhosis-drugs-market); however, no fundamental treatment for the disease has been developed to date. To overcome or prevent liver cirrhosis, it is essential to elucidate the molecular mechanisms by which fibrosis begins and progresses.

Hepatic stellate cells (HSCs) are a major source of myofibroblasts in the fibrotic liver. The activation of HSCs by fibrosis-related signals, such as transforming growth factor β (TGFβ), induces their trans-differentiation into myofibroblasts, which exacerbate fibrosis by promoting extracellular matrix deposition [3–6].

Retinol (vitamin A), a common component of a wide variety of nutritional supplements, is a signaling substance that plays an essential role in the body. Under normal conditions, retinol is stored almost in HSCs [7], but is mobilized toward extrahepatic storage sites during the progression of liver injuries, particularly those leading to fibrosis and cirrhosis [8]. However, the mechanism by which retinol is released from activated HSCs and affects hepatocytes has not been characterized.

A number of studies have reported contradictory correlations between retinol intake and liver disease. Whereas some studies found that retinol suppresses the progression of fibrosis and extracellular matrix accumulation, others reported opposing effects. Hyper-vitaminosis A is not considered a common condition but can lead to hepatic toxicity and cirrhosis in severe circumstances [9–11]. Current knowledge of the role of retinol in HSC activation and liver fibrosis is limited and requires further clarification.

STRA6 (Stimulated by retinoic acid 6) is a retinol-induced transmembrane protein [12–14], which plays a major role in retinol uptake in the eye [15]. In our previous study, we demonstrated that a reduced level of transcriptional intermediary factor 1γ (TIF1γ) leads to activation of HSCs [16] and the loss of stored retinol. In the current study, we demonstrate that STRA6 is regulated by TIF1γ and is responsible for the release of retinol from HSCs. In addition, we examined how this released retinol affects hepatocytes leading to fat deposition and liver fibrosis.

## Results

### Retinol released from HSCs promotes the accumulation of triglyceride in hepatocytes

**Fig. 1:**
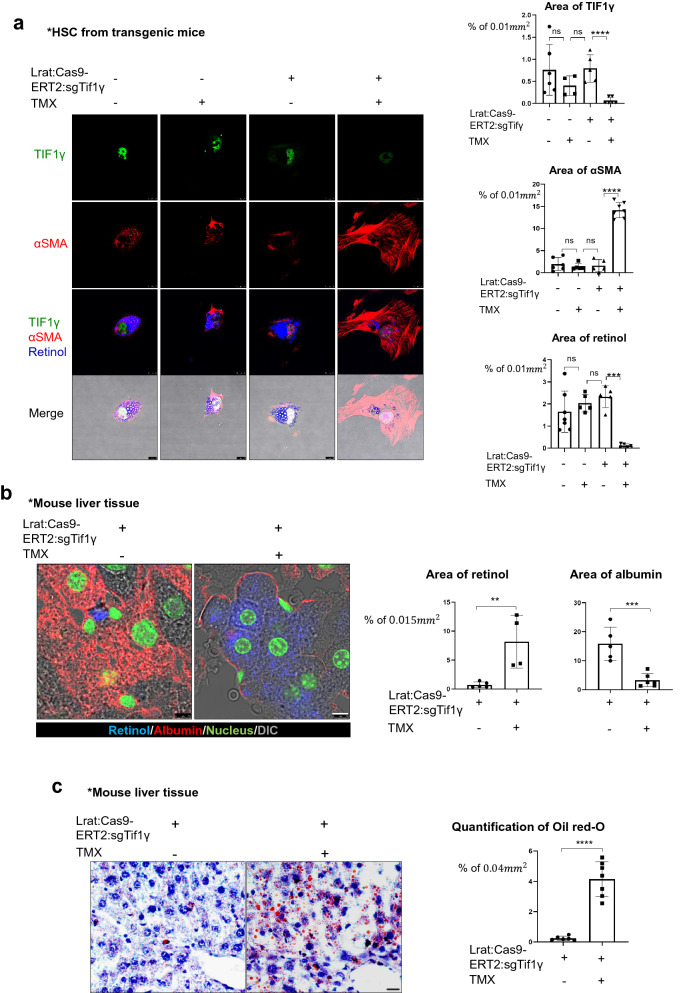

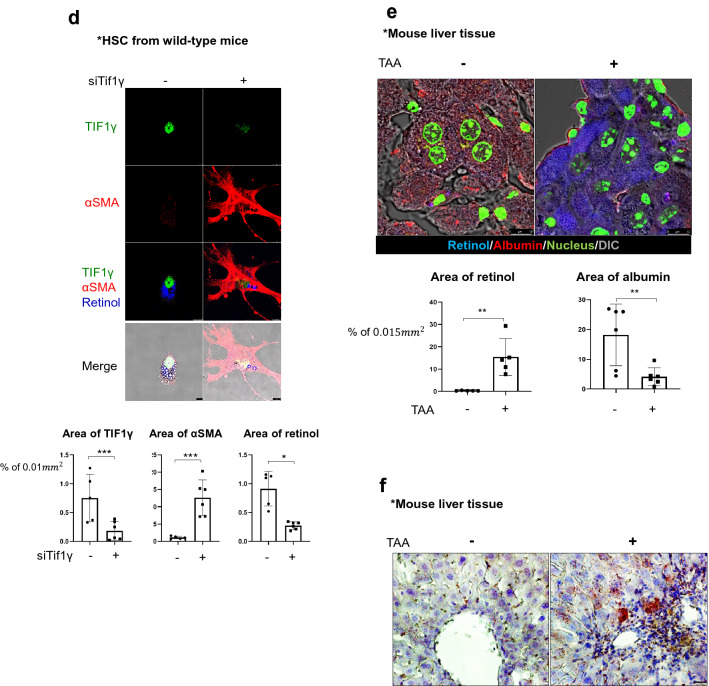
The fibrotic liver undergoes a loss of retinol from HSCs and an accumulation of triglyceride in hepatocytes. **a** Detection of retinol, TIF1γ, and αSMA in primary hepatic stellate cells from Lrat:Cas9-ERT2: sgTif1γ mice treated with or without 10 nM TMX in DMSO for 3 days. The control group was treated with DMSO only. TIF1γ is shown in green, αSMA is shown in red, and retinol is shown in blue. TIF1γ or αSMA-positive area % per 0.01 mm^2^ were quantified in five images each group using Image J program. Reproducible result from two independent experiments was shown. ***P < 0.001, ****P < 0.0001, ns; non-significant. Scale bar: 10 μm. **b** Detection of retinol (blue) and albumin (red) in the liver tissue of normal and Lrat:Cas9-ERT2: sgTif1γ mice treated with TMX in DMSO. The nucleus was stained with SYTOX green. DMSO only in TG, n = 4; TMX in TG, n = 4, complied from two experiments. Retinol or albumin-positive area % per 0.015 mm^2^ were quantified using Image J program. **P < 0.01, ***P < 0.001. Scale bar: 5 μm. **c** Oil red-O staining of triglyceride distribution in the liver of Lrat:Cas9-ERT2: sgTif1γ mice. The nuclei were stained with hematoxylin. DMSO only in TG, n = 4; TMX in TG, n = 4, complied from two experiments. Oil-red-O-positive area % per 0.04 mm^2^ were quantified using Image J program. ****P < 0.0001. Scale bar: 2 μm. **d** Detection of retinol, TIF1γ, and αSMA in primary hepatic stellate cells from normal mice treated with or without siTif1γ. TIF1γ is shown in green, αSMA is shown in red, and retinol is shown in blue. TIF1γ or αSMA-positive area % per 0.01 mm^2^ were quantified using Image J program. Reproducible result from two independent experiments was shown. *P < 0.05, ***P < 0.001, Scale bar: 10 μm. **e** Detection of retinol (blue) and albumin (red) in the liver tissue of normal and treated with TAA (n = 4 each group, two experiments). The nucleus was stained with SYTOX green. Retinol or albumin-positive area % per 0.015 mm^2^ were quantified using Image J program. **P < 0.01, Scale bar: 5 μm. **f** Oil red-O staining of triglyceride distribution in the normal and TAA-induced mouse liver (n = 4 each group, two experiments). Scale bar: 2 μm

To know the association between liver fibrosis driven by HSCs and intracellular amount of retinol in HSCs, we isolated primary HSCs from Lrat-specific Tif1γ knock-out transgenic mice (Lrat:Cas9-ERT2: sgTif1γ) [16] and cultured them for 3 days in the presence of 10 nM tamoxifen (TMX) to reduce TIF1γ levels, a negative regulator against fibrosis. Immunofluorescent imaging confirmed the successful knock-down of TIF1γ and an increase in the level of alpha-smooth muscle actin (αSMA), a marker of HSC activation (Fig. 1a). Under this situation, the level of retinol autofluorescence in the HSCs [17], detected using a violet 405 nm laser, was reduced (Fig. 1a).

We hypothesized that the retinol released from HSCs under low TIF1γ conditions would have an effect on the neighboring hepatocytes. To test this hypothesis, we examined the autofluorescence of retinol and the immunofluorescence of albumin, a functional marker of hepatocytes, in Lrat:Cas9-ERT2: sgTif1γ mice treated with or without TMX. In the untreated mice liver, retinol was observed only in HSCs, whereas it was spread throughout the hepatocytes in the TMX-treated mice liver (Fig. 1b). Consistent with the suggestion that retinol can have adverse effects on the liver, triglyceride was accumulated in the hepatocytes of TMX-treated Lrat:Cas9-ERT2: sgTif1γmice (Fig. 1c). Additionally, high mobility group box 1(HMGB1) was decreased in hepatocytes of TMX-treated Lrat:Cas9-ERT2: sgTif1γ mice, which was confirmed by retinol treated HepG2 cells as hepatocyte (Additional file 1: Fig. S1a, b). Secreted HMGB1, a signal of cell death [18], was increased according to retinol treatment to HepG2 (Additional file 1: Fig. S1B). To confirm that these phenomena are a common response to liver injury leading to fibrosis, we performed similar analysis in wild mice and a thioacetamide (TAA)-induced fibrosis model. As expected, know-down of Tif1γ in HSC from wild mice liver showed loss of retinol with increased αSMA (Fig. 1d). Moreover, the distributions of retinol (Fig. 1e) and triglyceride (Fig. 1f) in the TAA-induced fibrotic liver were similar to those in the TMX-treated Lrat:Cas9-ERT2: sgTif1γ liver. Taken together, these findings provide clues of association between retinol released from HSCs and the development of lipogenesis in hepatocytes during liver fibrosis after injury.

### Retinol increases the expression levels of de novo lipogenesis-related genes in hepatocytes

**Fig. 2:**
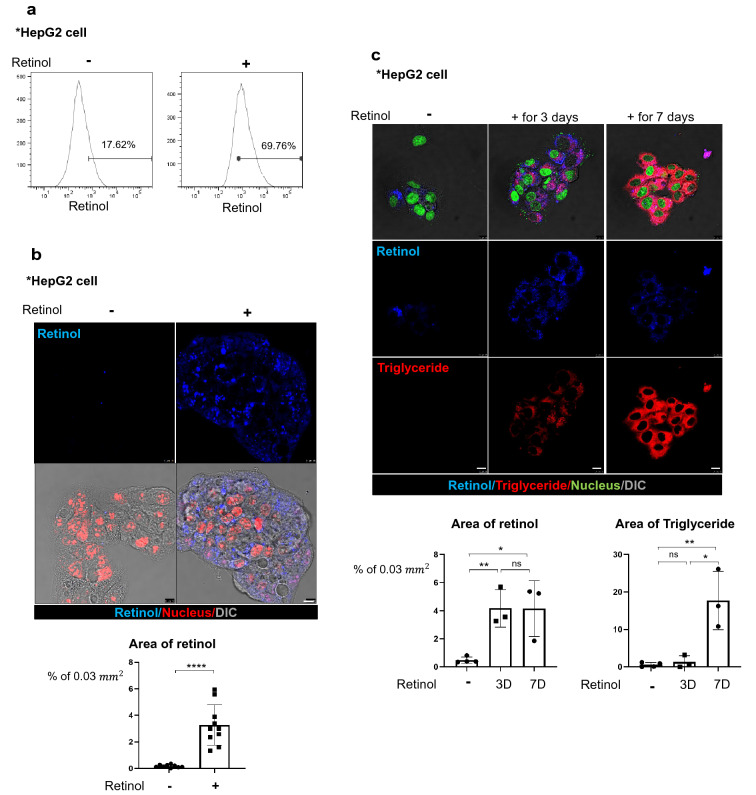

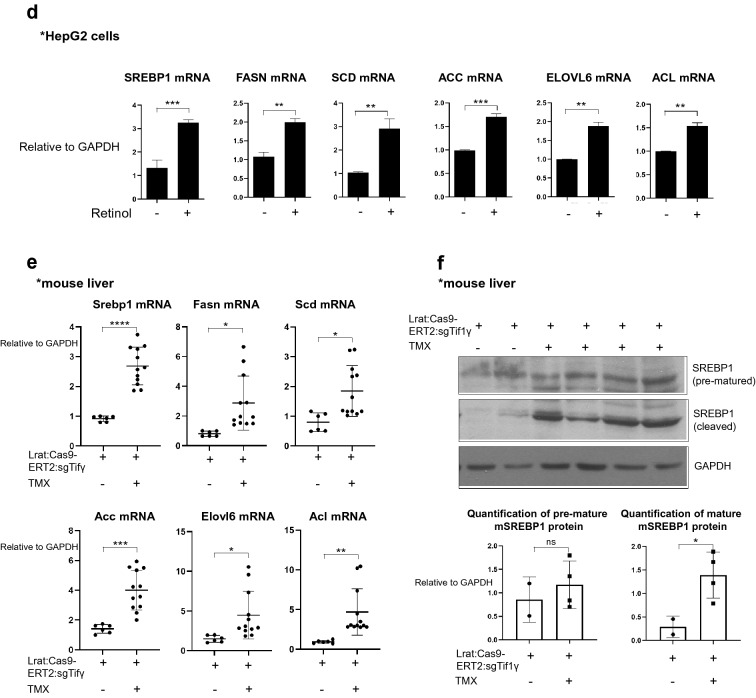
Retinol promotes the production of triglyceride in hepatocytes by increasing the expression levels of lipogenesis-related genes in vitro and in vivo. **a** Flow cytometric analysis using the autofluorescence of retinol. HepG2 cells were treated with or without 1 μm retinol (Ret) for 24 h, and were detected using a 450/50 filter and a 405 nm violet laser. HepG2 treated only with DMSO was used as the negative group, and retinol positivity was analyzed based on hoechst33258, which includes the excitation emission wavelength range of retinol. Reproducible result from two independent experiments was shown. **b** The accumulation of retinol (Ret; blue) in HepG2 cells treated with or without 1 μm retinol for 24 h. The nuclei were stained with SYTOX orange (red). Retinol-positive area % per 0.03 mm^2^ were quantified using Image J program. ****P < 0.0001 Scale bar: 10 μm. DIC, differential interference contrast showing the bright phase image of the samples. Reproducible result from three independent experiments was shown. **c** The distributions of retinol (Ret; blue) and triglyceride (red) in HepG2 cells treated with or without 1 μM retinol for 3 or 7 days. The retinol and its culture media were treated freshly every day because retinol is degraded easily.The nuclei were stained with SYTOX green. Retinol or triglyceride-positive area % per 0.03 mm^2^ were quantified using Image J program. Reproducible result from three independent experiments was shown. *P < 0.05, **P < 0.01, ns; non-significant. Scale bar: 10 μm. D. The expression levels of lipogenesis-related genes in HepG2 cells treated with or without 1 μm retinol in DMSO as negative control. hSREBP1: sterol regulatory element-binding transcription factor 1; hFASN: fatty acid synthase; hSCD: stearoyl-CoA desaturase-1; hACC: acetyl CoA carboxylase; hACL: ATP citrate lyase; hELOVL: ELOVL fatty acid elongase 6. Reproducible result from three independent experiments was shown. **P < 0.01, ***P < 0.001. **e** The expression levels of lipogenesis-related genes in the livers of Lrat:Cas9-ERT2: sgTif1γ mice treated with or without TMX (Lrat:Cas9-ERT2: sgTif1γ/Control n = 2, Lrat:Cas9-ERT2: sgTif1γ/TMX n = 3). The experiments were performed in triplicate. Unpaired Student’s t-tests were performed in Prism8. *P < 0.05, **P < 0.01, ***P < 0.001, ****P < 0.0001. F. The expression levels of premature and cleaved SREBP1 in the livers of Lrat:Cas9-ERT2: sgTif1γ mice treated with or without TMX(each line represented independent mouse). The expression level of GAPDH was used as a loading control. *P < 0.05, ns; non-significant

To determine the relationship between the distribution of retinol and the accumulation of triglyceride in hepatocytes, we examined the response of the human HepG2 cell line to retinol. Flow cytometry analyses revealed that the percentage of retinol-positive HepG2 cells in the total cell population increased from 17.6% to 69.8% following treatment with 1 µM retinol compared to DMSO as its vehicle for 1 day (Fig. 2a). Fluorescence analyses revealed that retinol was located in the cytoplasm of HepG2 cells (Fig. 2b). Following prolonged treatment of the cells with retinol, the cytoplasmic retinol signal increased after 3 days but then decreased after 7 days. By contrast, the level of triglyceride in the cells, detected using BODIPY, increased progressively on days 3 and 7 (Fig. 2c).

Given that HepG2 is a cancer-derived cell line, we also examined the effect of retinol on HepaRG cells that can differentiate to human hepatocyte-like cells, with characteristics similar to those of human primary hepatocytes [19]. In HepaRG cells treated with retinol for 7 days, we observed a “pushed aside nucleus” and balloon-like morphology caused by the storage of triglyceride in the cytoplasm (Additional file 1: Fig. S2).

In view of a previous report that lipogenesis in the liver could promote de novo lipogenesis [20], we examined the expression levels of six de novo lipogenesis-related genes in HepG2 cells treated with or without 1 µM retinol for 3 or 7 days (Fig. 2d). The mRNA levels of all six lipogenesis-related genes were elevated after retinol treatment. Those examined included sterol regulatory element-binding transcription factor 1 (SREBP1), a critical transcription factor that regulates lipogenesis-related genes; fatty acid synthase (FASN); ATP citrate lyase (ACL); acetyl CoA carboxylase (ACC); stearoyl-CoA desaturase-1 (SCD); and ELOVL fatty acid elongase 6 (ELOVL6). In addition, the expression levels of all six genes were higher in the livers of TMX-treated Lrat:Cas9-ERT2: sgTif1γ mice than in those of untreated Lrat:Cas9-ERT2: sgTif1γ mice (Fig. 2e). Finally, we found that knock-down of TIF1γ by TMX treatment in Lrat:Cas9-ERT2: sgTif1γ mice increased the level of cleaved (activated) SREBP1 in the liver (Fig. 2f). Overall, these findings suggest that retinol released from HSCs during process of knock-down of TIF1γ and myo-fibroblastic activation of HSCs after injury can promote triglyceride accumulation in hepatocytes via de novo lipogenesis.

### Retinol induces de novo lipogenesis in hepatocytes via the retinoic acid receptor

**Fig. 3:**
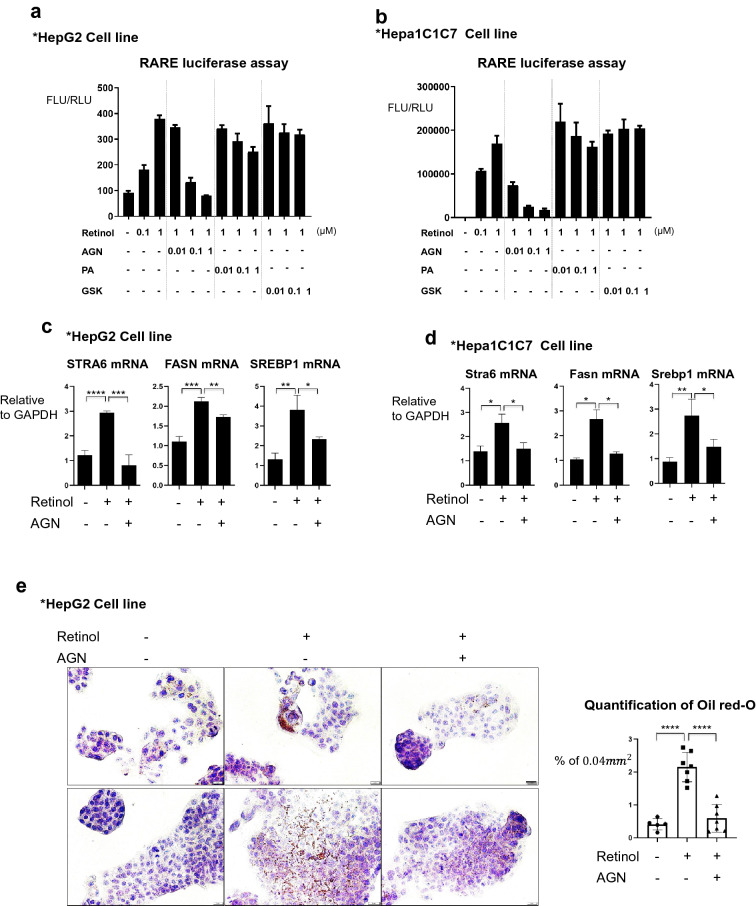
The RAR mediates increases in the expression levels of lipogenesis-related genes in response to retinol. **a** Luciferase activity of the pGL3-RARE construct in transfected HepG2 cells. The cells were treated with or without the indicated concentrations of retinol, the RAR antagonist AGN193109 (AGN), the RXR antagonist PA452 (PA), and the LXR blocker GSK2033 (GSK) in DMSO as its vehicle for 24 h. FLU: Firefly Luciferase Unit; RLU: Renilla Luciferase Unit. Reproducible result from three independent experiments was shown. **b** The luciferase activity of the pGL3-RARE construct in Hepa1C1C7 cells. The cells were treated as described for A. Reproducible result from three independent experiments was shown. C. The expression levels of the mRNAs encoding human STRA6, FASN, and SREBP1 in HepG2 cells treated with or without 1 μM retinol and/or 100 nM AGN in DMSO for 1 day. The experiments were performed in triplicate. Unpaired Student’s t-tests were performed in Prism8. *P < 0.05, **P < 0.01, ***P < 0.001, ****P < 0.0001. D. The expression levels of the mRNAs encoding mouse STRA6, FASN, and SREBP1 in Hepa1C1C7 cells treated with or without 1 μM retinol and/or 100 nM AGN for 1 day. The experiments were performed in triplicate. Unpaired Student’s t-tests were performed in Prism8. **P < 0.01, ***P < 0.001, ****P < 0.0001. **e** Oil red-O staining showing the accumulation of triglyceride in HepG2 cells treated with or without 1 μM retinol and/or 100 nM AGN for 3 days. Oil-red-O-positive area % per 0.04 mm^2^ were quantified using Image J program. Reproducible result from two independent experiments was shown. ****P < 0.0001. The nuclei were stained with hematoxylin. Scale bar: 2 μm

SREBP1 mediates the retinoid-dependent increase in FASN expression in hepatocytes [21]. In addition, there have been suggestions of LXR linked to lipogenesis [22] and RARE / LXRE binding to the existence of the sites on the promoter of *SREBP1* [23]. Therefore, we hypothesized that the activation of de novo lipogenesis in hepatocytes by retinol may be mediated through RAR. To examine this possibility, we transfected HepG2 cells with the pGL3-RARE plasmid, a luciferase reporter construct containing a synthetic retinoic acid receptor (RAR)-specific response element (RARE), and treated them with 0.1–1 µM retinol (Fig. 3a). Subsequently, 1 µM retinol was selected as an effective dose that induced a detectable luciferase signal. The RAR antagonist AGN193109 attenuated the increase in luciferase signal caused by 1 µM retinol (Fig. 3a). By contrast, this inhibitory effect was not seen when the cells were treated with GSK2033, an antagonist of the liver X receptor (LXR) which binds the RAR in the liver [22], or with PA452, an antagonist of the retinoid X receptor (RXR) which acts as a binding partner of the RAR [24] (Fig. 3a). Similar results were obtained when the experiment was repeated using mouse hepatocarcinoma Hepa1C1C7 cells (Fig. 3b), confirming that AGN193109 is the only specific blocker of retinol/RAR signal both in mouse and human hepatocytes.

Next, we examined the effect of retinol on mRNA levels of lipogenesis-related genes in human HepG2 and mouse Hepa1C1C7 cells. Retinol significantly induced these genes, which was significantly attenuated by AGN193109 (Fig. 3c, d). Furthermore, in oil red-O staining of HepG2 cells, the retinol induced accumulation of triglycerides, which was attenuated by blocking the RAR by AGN193109 (Fig. 3e).

### Retinol is released from activated HSCs via STRA6

Next, we performed a mechanistic study in vitro to determine how retinol is released from HSCs. To this end, the human hepatic stellate LX2 cell line was pretreated with retinol to ensure intracellular retinol loading. Immunofluorescence (Additional file 1: Fig. S3A) and FACS (Additional file 1: Fig. S3A) analyses confirmed that the retinol treatment increased the percentage of retinol-positive cells from 1.7% to approximately 80%. (Additional file 1: Fig. S2B). Subsequently, we examined the expression levels of several genes that are involved in the mobilization or metabolism of retinol in HSCs, such as, STRA6, adipose triglyceride lipase (ATGL), and Patatin-like phospholipase domain-containing protein 3 (PNPLA3) [25–27]. Interestingly, the expression level of the STRA6 mRNA was significantly increased by TGFβ1, the known activator of HSCs and inducer of fibrosis. Such induction of STRA6 was significantly prevented by treatment of the cells with hepatocyte growth factor (HGF), the known inhibitor of fibrosis [28], (Additional file 1: Fig. S3C). The expression levels of the mRNAs encoding ATGL and PNPLA3 were decreased slightly following treatment of LX2 cells with TGFβ1 (Additional file 1: Fig. S3C), which was not affected anymore by HGF. In view of our finding that loss of TIF1γ activates HSCs [16, 29], we also examined the effect of modulating TIF1γ levels on STRA6 expression in LX2 cells. Knock-down of TIF1γ by siRNA increased the expression level of the STRA6 mRNA, while overexpression of TIF1γ decreased it (Additional file 1: Fig. S3D).

**Fig. 4:**
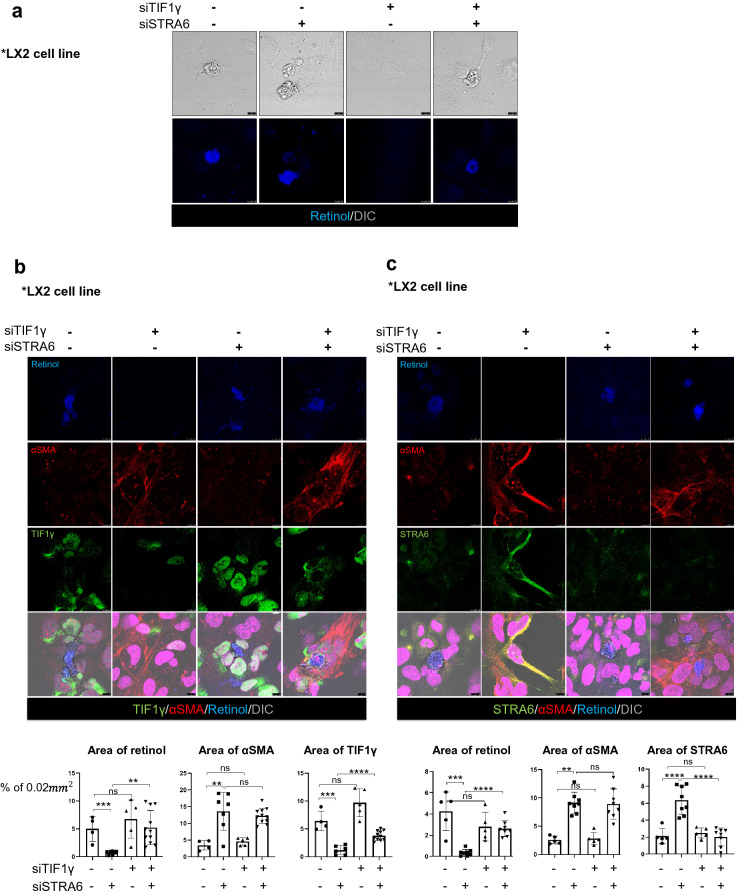
Knock-down of STRA6 inhibits the release of retinol from activated HSCs. **a** The LX2 cell line was treated with 1 μM retinol for 3 days following transfection with siRNAs targeting human TIF1γ and/or STRA6. The DIC channel (bright image) shows the cell morphology, and retinol autofluorescence is shown in blue. Reproducible result from two independent experiments was shown. Scale bar: 10 μm. **b,** **c** The experimental conditions were the same as described for A. Retinol autofluorescence is shown in blue, αSMA is shown in red, and TIF1γ (**b**) or STRA6 (**c**) is shown in green. The nuclei were stained with TO-PRO 3 (magenta). Reproducible result from two independent experiments was shown. Retinol or αSMA or TIF1γ-positive area % per 0.02 mm^2^ were quantified using Image J program. Reproducible result from three independent experiments was shown. **P < 0.01, ***P < 0.001, ****P < 0.0001, ns; non-significant. Scale bar: 10 μm

Next, we examined the effects of siRNA-mediated knock-down of TIF1γ or STRA6 on retinol levels in LX2 cells that were pretreated with retinol while siRNA were treated twice. Knock-down of TIF1γ made LX2 cells not maintain retinol loaded in cytoplasm leading to reduction of intracellular retinol, whereas knock-down of STRA6 had no effect (Fig. 4a). Subsequently, we treated the cells with retinol for 3 consecutive days and then co-transfected them with siSTRA6 and siTIF1γ. Retinol autofluorescence detected using a 405 nm laser is shown in blue, αSMA in the activated cells is shown in red, and TIF1γ (Fig. 4b) or STRA6 (Fig. 4c) is shown in green. The nuclei were stained with TO-PRO 3 (magenta). Reproducible results were obtained from two independent experiments. The knock-down of TIF1γ induced leakage of retinol from LX2 cells leading to reduction in the intracellular retinol, which was blocked by concurrent knock-down of STRA6 (Fig. 4b, c). Taken together, these findings indicate that retinol is released via STRA6 from activated HSCs with reduction of TIF1γ.

### Retinol induces STRA6 expression in hepatocytes during process of liver injury leading to fibrosis

**Fig. 5:**
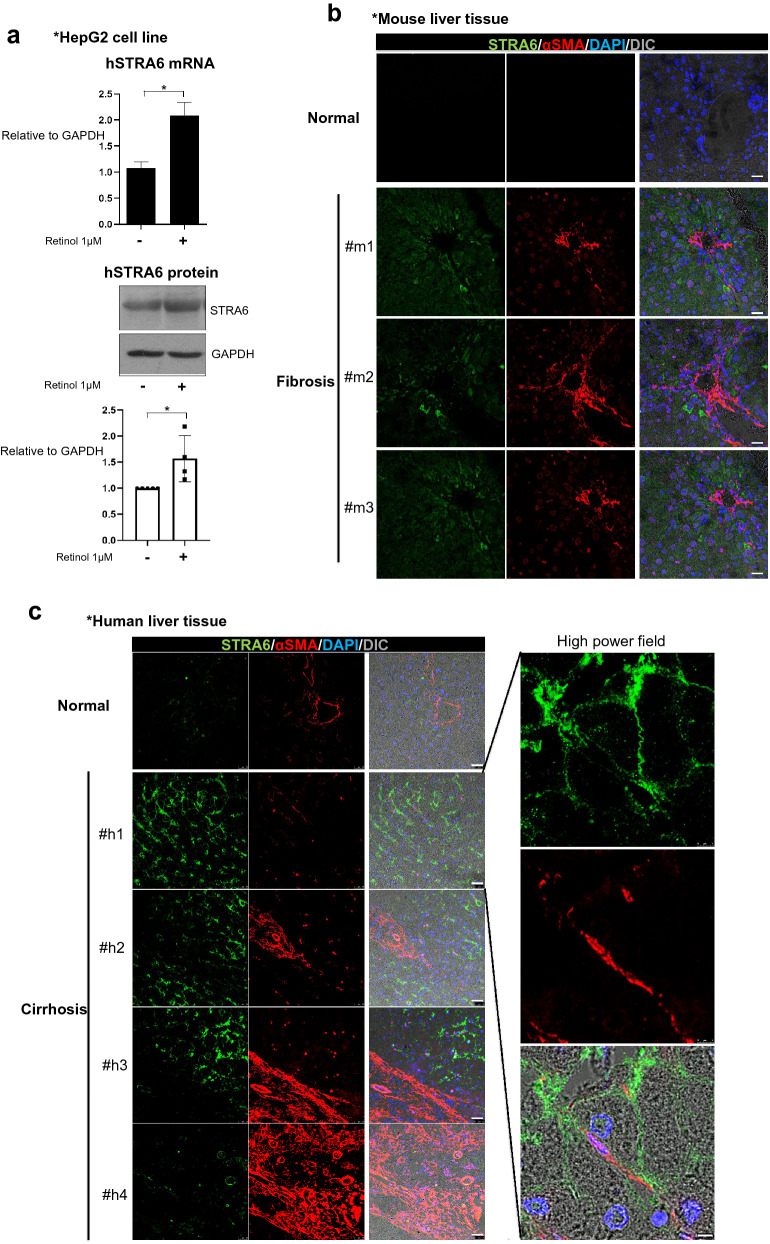

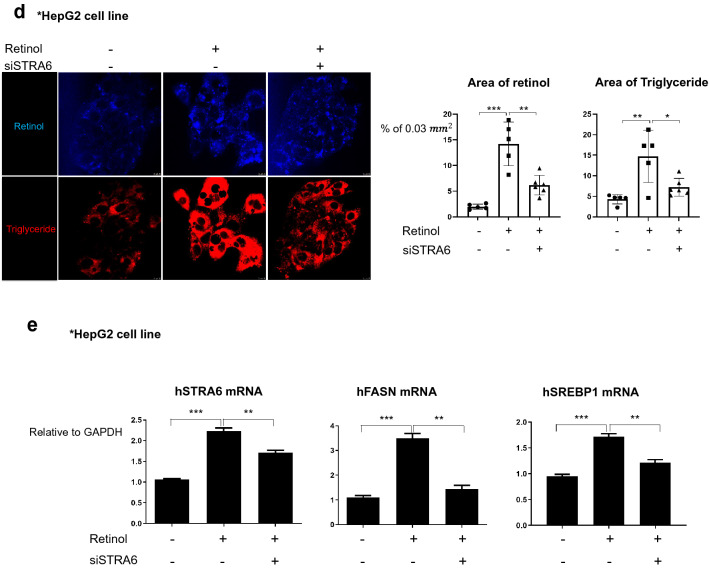
The expression of human and mouse STRA6 in vitro and in vivo. **a** HepG2 cells were treated with 1 μM retinol in DMSO for 1 day, and the expression levels of the human STRA6 mRNA and protein were determined. The expression level of GAPDH was detected as an endogenous control. The experiments were performed in triplicate. The quantification of western blot data was performed using Image J and unpaired Student’s t-tests were performed in Prism8. *P < 0.05.** b** The distributions of STRA6 and αSMA in normal (n = 3) and TAA-induced fibrotic mouse liver tissues (n = 3). STRA6 is shown in green, and αSMA is shown in red. The nuclei were stained with DAPI (blue). Scale bar: 25 μm. **c** The distributions of STRA6 and αSMA in normal (n = 3) and cirrhotic human liver tissues (n = 4). STRA6 is shown in green, and αSMA is shown in red. The nuclei were stained with DAPI (blue). Four samples in the cirrhosis group were analyzed independently, and representative data are shown in each panel. Scale bar of lower magnification: 25 μm; scale bar of higher magnification: 5 μm.** d** The accumulation of triglyceride in HepG2 cells treated with or without 1 μM retinol in DMSO for 24 h following transfection with a siRNA targeting human STRA6. Retinol autofluorescence is shown in blue, and triglyceride was stained using BODIPY (red). Retinol or triglyceride-positive area % per 0.03 mm^2^ were quantified five images using Image J program. Reproducible result from three independent experiments was shown. *P < 0.05, **P < 0.01, ***P < 0.001. Scale bar: 10 μm. **e** The expression levels of the mRNAs encoding human STRA6, FASN, and SREBP1 in HepG2 cells transfected with or without a siRNA targeting STRA6, and treated with or without retinol as described in** a**. The experiments were performed in triplicate. Unpaired Student’s t-tests were performed in Prism8. **P < 0.01, ***P < 0.001

Because STRA6 expression is induced in HSCs by TGFβ1 or knock-down of TIF1γ that controlled intracellular retinol in HSCs, we wondered whether retinol released from HSCs induces STRA6 expression on hepatocytes. STRA6 expression is reported to be very low in the liver (https://www.proteinatlas.org/ENSG00000137868-STRA6/tissue) [30]. Our first step was to determine whether the STRA6 protein could be detected by a specific antibody in western blotting. To this end, myc-tagged human STRA6 was overexpressed in 293 T cells, and the cells were treated with or without retinol. Western blotting analyses revealed that exogenous STRA6 was detected in 293 T cells using an anti-myc or anti-STRA6 antibody, both in the presence and absence of retinol (Additional file 1: Fig. S4). Because HepG2 cells does not express STRA6 on the ground state,we mimicked the in vivo situation by pretreating HepG2 cells with retinol, and found that STRA6 expression was induced both at the mRNA and protein level compared to the negative cotnrol DMSO only treated (Fig. 5a). In addition, STRA6 expression was detected widely in the hepatocytes of the TAA-induced fibrotic mouse liver but was not detected in the DMSO as its vehicle treated normal liver (Fig. 5b). Such findings were observed also in human liver; no expression of STRA6 in hepatocytes from normal person, while high expression of STRA6 in the surviving hepatocytes in cirrhotic liver from patients (Fig. 5c). Retinol treatment increased the levels of retinol and triglyceride in HepG2 cells, which was attenuated by knock-down of STRA6 in cells expressing siSTRA6 (Fig. 5d). Transfection of HepG2 cells with siSTRA6 also attenuated the retinol-induced increases in the levels of the mRNAs encoding FASN and SREBP1 (Fig. 5e). Overall, these findings indicate that retinol induces STRA6 expression in hepatocytes, leading to induction of lipogenesis genes and deposition of triglyceride in hepatocytes.

### Specific gene targeting on Stra6 in HSCs using vitamin/liposome complex prevents the accumulation of retinol and triglyceride in hepatocytes, leading to reduction of liver fibrosis and injury in mouse model

**Fig. 6:**
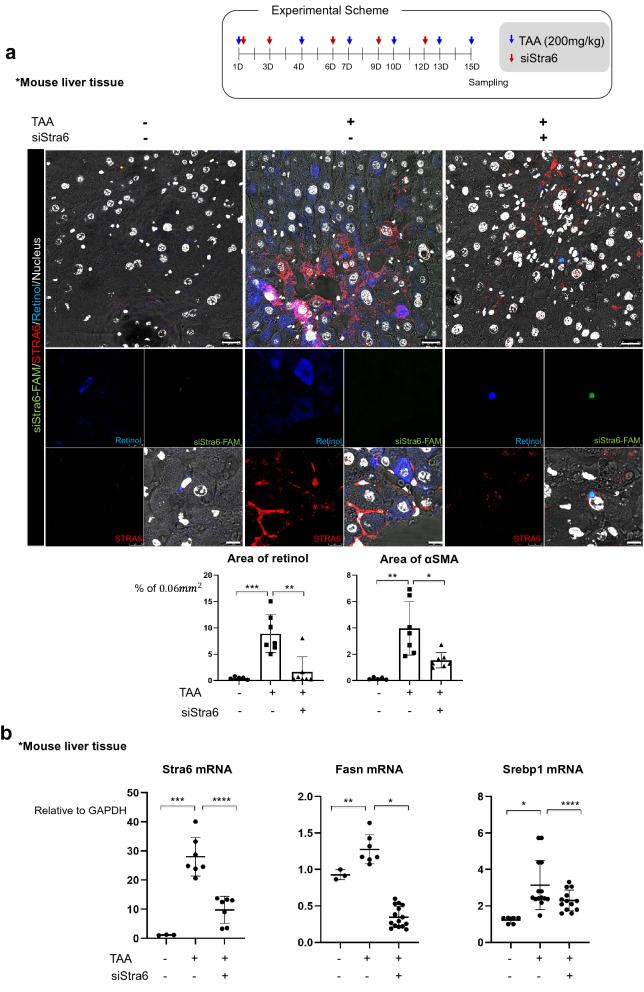

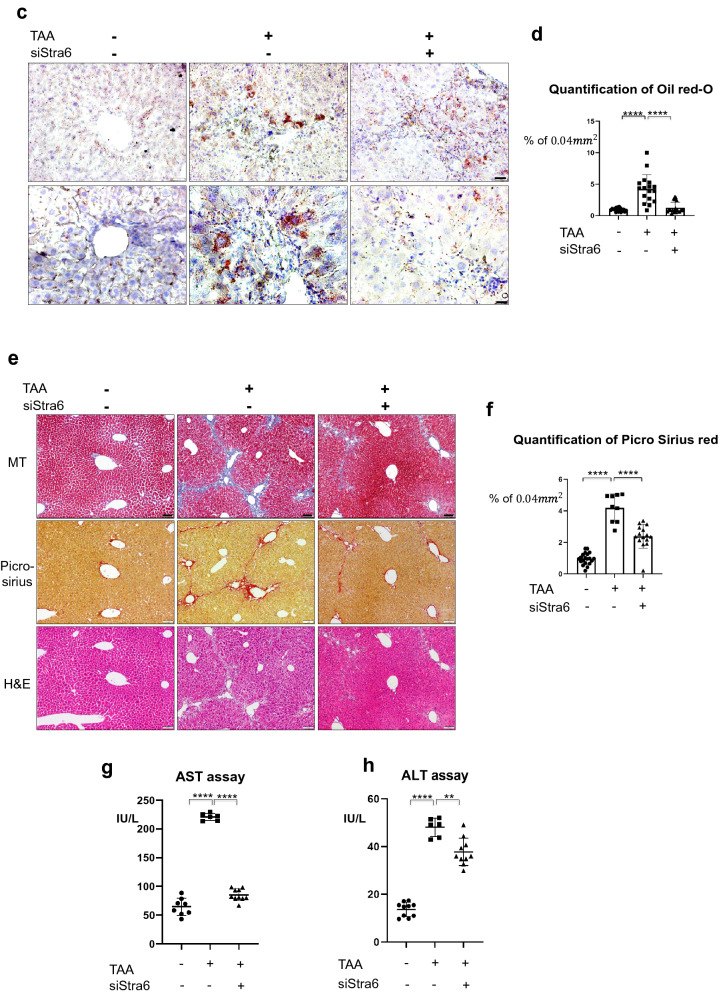
Knock-down of Stra6 attenuates liver fibrosis in vivo. **a** Delivery of a FAM-tagged siRNA targeting Stra6 to the normal or TAA-treated mouse liver using a vitamin A-liposome complex. The FAM-tagged siRNA targeting Stra6 is shown in green, STRA6 is shown in red, retinol is shown in blue, and the nuclei are shown in white. VitaminA-liposome complex only-treated group: n = 3; vitaminA-liposome complex and TAA-treated group: n = 3; TAA/siStra6 group: n = 5. The experiments were performed three times independently. Retinol or αSMA-positive area % per 0.06 mm^2^ were quantified using Image J program in five pictures for each group. Scale bar of lower magnification: 25 μm; scale bar of higher magnification: 5 μm. **b** The effects of siRNA-mediated knock-down of Stra6 on the expression levels of the mRNAs encoding STRA6, FASN, and SREBP1 in normal and TAA-treated mouse liver tissues. VitaminA-liposome complex only-treated group: n = 3; vitaminA-liposome complex and TAA-treated group: n = 3; TAA/siStra6 group: n = 5. The experiments were performed three times independently. P-values were determined using unpaired Student’s t-tests in Prism8. *P < 0.05, **P < 0.01, ***P < 0.001, ****P < 0.0001.** c** Oil red-O staining showed the accumulation of triglyceride in TAA- and siStra6-treated mouse liver samples. VitaminA-liposome complex only-treated group: n = 3; vitaminA-liposome complex and TAA-treated group: n = 3; TAA/siStra6 group: n = 5. The experiments were performed three times independently. Scale bar of the upper panel: 5 μm; scale bar of the lower panel: 2 μm. **d** Quantification of the oil red-O staining shown in (**c**). VitaminA-liposome complex only-treated group: n = 3; vitaminA-liposome complex and TAA-treated group: n = 3; TAA/siStra6 group: n = 5. Five pictures of each sample were analyzed using ImageJ. P-values were determined using unpaired Student’s t-tests in Prism8. ****P < 0.0001. **e** MT staining of the extracellular matrix and picro-sirius staining of collagen in the VitaminA-liposome complex only-, vitaminA-liposome complex and TAA-treated, and TAA plus siStra6-treated groups. Hematoxylin and eosin (H&E) staining was also performed. Scale bar: 10 μm. **f** Quantification of the picro-sirius red staining shown in **e** VitaminA-liposome complex only-treated group: n = 3; vitaminA-liposome complex and TAA-treated group: n = 3; TAA/siStra6 group: n = 5. Four pictures of each sample were analyzed using ImageJ. P-values were calculated using unpaired Student’s t-tests in Prism8. ****P < 0.0001. **g**, **h** The levels of aspartate aminotransferase (**g**) and alanine aminotransferase (**h**) in mouse serum. Duplicates of each sample were performed. VitaminA-liposome complex only-treated group: n = 3; vitaminA-liposome complex and TAA-treated group: n = 3; TAA/siStra6 group: n = 5. P-values were calculated using unpaired Student’s t-tests in Prism8. **P < 0.01, ****P < 0.0001

Given our results indicating that STRA6 plays a role in promoting lipogenesis in hepatocytes via uptake of retinol released from HSC during fibrosis, we hypothesized that suppression of STRA6 would repress liver fibrosis. Since HSCs can take up and store retinol, the vitamin A-liposome complex is a well-established method of targeted delivery to these cells [31]. A FAM-tagged siRNA targeting Stra6 was delivered to the mouse liver using a vitamin A-liposome complex, and the distributions of FAM and retinol were detected. In the TAA-induced fibrotic liver, STRA6 expression was induced in both HSCs and hepatocytes, and retinol was dispersed in hepatocytes. By contrast, dispersed retinol was not detected around HSCs displaying a FAM signal, indicating successful uptake of the siRNA and knock-down of Stra6 (Fig. 6a). Knock-down of Stra6 also reduced the expression levels of Fasn and Srebp1 (Fig. 6b), as well as the accumulation of triglyceride (Fig. 6c, d). Furthermore, MT staining of the extracellular matrix and picro-sirius staining of collagen revealed that the fibrotic area of the mouse liver was reduced following knock-down of Stra6 (Fig. 6e, f). Finally, the liver enzyme significantly elevated after liver injury by TAA, which was effectively prevented by knock-down of Stra6 (Fig. 6g, h).

## Discussion

### The expression of STRA6 in HSCs and hepatocytes in the fibrotic liver

Stra6 was reported in 1995 as an unknown functional gene that is transcriptionally upregulated by retinoic acid [32]. Since its molecular identification as a retinol binding protein (RBP) receptor, the increasing evidences have indicated that STRA6 preferentially mediates the uptake of all-trans-retinol-bound RBP [13]. Consistently, experimental evidence suggested that the existence of a pore or “passageway” in STRA6′s transmembrane domains for transport vitamin A [15]. The function and regulation of STRA6 in the retina has been studied extensively, but its regulation in the fibrotic liver has remained unclear [15]. Here, we found that stored retinol is released by activated HSCs via STRA6 and then taken up into hepatocytes, again via STRA6. In addition, we found that TIF1γ suppresses STRA6 expression and thus inhibits release of retinol from HSCs to surrounding hepatocytes, leading to the normal, non-fatty, and non-fibrotic liver. These results suggest that TIF1γ and STRA6 may be a good target to prevent activation HSC and liver fibrosis as well as retinol-induced hepatocyte fat deposition and fatty liver.

In this study, we made an important progress in experimental technique by optimizing the culture system to observe retinol deposition in the LX2 cell line. LX2 is a primary human hepatic stellate cell line immortalized via transfection of the SV40T antigen [33, 34]. Although LX2 cell line historically has been sold as a commercial product, it has been difficult to observe retinol droplets in LX2 cells under ordinary culture conditions. Here, we observed retinol droplets in the cells after 3 days of treatment with retinol and thus able to dissect the detailed mechanism how retinol moves from HSCs to hepatocytes leading lipogenesis, hepatotoxicity, and liver fibrosis.

### Retinol-induced de novo lipogenesis in hepatocytes could be related to hepatic steatosis and fibrosis

A correlation between steatosis and fibrosis has been reported in patients with liver disease [35–37]. Our results presented here, showing that retinol released from HSCs early after liver injury which results in fibrosis later led to lipogenesis in hepatocytes, support a positive correlation between fibrosis and lipogenesis in the liver [36]. The fact that the major transcription factors involved in de novo lipogenesis [20] are regulated by retinol via STRA6 suggests that steatosis arising from diet-induced obesity might be caused by the hepatocyte accumulation of retinol which was released via STRA6 from activated HSC. In addition, the newly synthesized triglyceride may cause oxidative stress during fibrosis and may lead to cell death [38]. Overall, we suggest that lipogenesis in hepatocytes, initiated by retinol, increases the possibility of developing hepatic steatosis and exacerbates fibrosis.

### Several strategies including suppression of STRA6 in HSCs could be promising therapeutic tools for fatty liver disease and liver cirrhosis

Here, we found that siRNA-mediated knock-down of Stra6 suppressed the early development of liver fibrosis in TAA-liver injury mouse model (Fig. 6). Notably, delivery using the vitamin A-liposome complex allowed specific targeting of the siRNA of STRA6 to HSCs, and could be used as a strategy to prevent the initial development of fat deposition and damage in hepatocytes and liver fibrosis by knocking down STRA6 in these cells (Fig. 6).

We reported in previous paper [16] that the first step to liver fibrosis in response to injury is the TGFβ1-induced TIF1γ suppression in the activated HSC. In the current manuscript, we found the subsequent mechanisms how the activated HSCs exert damage on hepatocytes. First, the suppressed TIF1γ in HSC increases the STRA6 expression, leading to leakage of retinol from HSC toward surrounding hepatocytes. Second, the leaked retinol is taken up by hepatocytes through STRA6 channel and then induces SREBP1 and STRA6 expression through RAR/LXR transcription factor complex. Finally, SREBP1 induces a cluster of lipogenesis genes and fat deposition in hepatocytes, while STRA6 expression enhances uptake of retinol released from HSC and establishes the vicious cycle, leading fatty liver disease and liver cirrhosis. Thus we are able to suggest several steps of targets to prevent liver fat deposition and fibrosis, for example, (1) supplementation of TIF1γ gene in HSC, and (2) knock-down of STRA6 in HSC and hepatocytes.

In conclusion, fatty liver disease and cirrhosis in response to injury may be induced by complex interplay between HSCs and hepatocytes by transport of retinol between these cells through STRA6. Thus we have to pay attention to the complex pathogenic mechanisms involving multiple cell types in order to develop the effective therapeutics for fatty liver disease and cirrhosis.

## Materials and methods

### Cell culture

LX2 cells were purchased from Merck Millipore (Cat. SCC064, Merch Millipore). LX2 cells were grown in LX2 complete medium at 37 °C in a humidified incubator with 5% CO_2_. LX2 complete medium comprised high-glucose DMEM supplemented with GlutaMAX (Gibco, Grand Island, NY, USA), 2% fetal bovine serum (FBS) (Cat. 16,000, Gibco), and 1% (v/v) penicillin/streptomycin (Cat. 15070063, Gibco). HepG2 cells were grown in high-glucose DMEM supplemented with 10% FBS and 1% (v/v) penicillin/streptomycin (Gibco). HepaRG cells (Cat. HPRGC10) were purchased from Thermo Fisher Scientific (Waltham, MA, USA) and were maintained in William’s medium E (Cat. 12551032, Thermo Fisher Scientific) containing 1 × GlutaMAX (Cat. 35050061, Thermo Fisher Scientific) and 10% FBS (Cat. HPRG620, Thermo Fisher Scientific).

### Mouse fibrosis model

All experiments were approved by the Institutional Animal Care and Use Committee in Seoul National University Hospital (SNUH-IACUC, No 19-0177-S1A0) and animals were maintained in the facility accredited AAALAC International (#001169) in accordance with Guide for the Care and Use of Laboratory Animals 8th edition, NRC (2010). Male 12–13-week-old BALB/c-nude mice weighing 20–25 g were used for experiments. To induce liver fibrosis, mice were given an intraperitoneal injection of TAA at a dose of 200 mg/kg (Sigma-Aldrich, St. Louis, MO, USA) or phosphate-buffered saline (PBS, control) three times a week for 1–3 weeks. Generation of the TIF1γ-knock-out mice has been described previously [16]. Transgenic mice expressing Lrat:Cas9-ERT2: sgTif1γ were generated and interbred under pathogen-free conditions at Macrogen (Seoul, Korea). All manipulations were conducted with the approval of the Macrogen IACUC. The siRNA targeting mouse Stra6 was delivered using vitamin A-coupled liposomes, as reported previously. The sequence of the siRNA was as follows: sense, [FAM]CAGCUACUCCGAGAAGUAU = tt; antisense, AUACUUCUCGGAGUAGCUG = gt.

### Generation of TMX-inducible TIF1γ-knockout mice

Transgenic (TG) mice expressing pLRAT:Cas9-ERT2: sgTIF1γ were generated and interbred in pathogen-free conditions at Macrogen (Seoul, Korea). Briefly, the pLRAT:Cas9-ERT2: sgTIF1γ DNAs were linearized, and microinjected into one cell of embryo using standard microinjection procedures (Macrogen, Seoul, Korea). Fourteen to sixteen injected one-cell-stage embryos were transplanted into the oviducts of pseudopregnant recipient mice (ICR). Founders were identified by PCR (using primers specific to Cas9-ERT2 (F: 5′-TGCTACAGAACAGTTGCAGCC-3′, R: 5′-ACCTTGTACTCGTCGGTGATC-3′) and TIF1γ gRNAs (U6-F: 5′-GTCGACGAGGGCCTATTTCCCATGATT-3′, gRNA1-R: 5′-TCGTCGGGCCCAGCCGCACC-3′, gRNA2-R: 5′-GTATGTCGTCAAGAATGTAG-3′, and gRNA 3-R: 5′-ACTGCACTTGCATTATCTTC-3′) and generated to F2, male 12-week-old mice were used for experiments.

### Antibody, siRNA, overexpression clone and chemical

A rabbit polyclonal anti-TIF1γ antibody (Cat. ab47062, Abcam) was used for immunofluorescence and western blotting analyses, and a different rabbit polyclonal anti-TIF1γ antibody (Cat. ab84455, Abcam) was also used for western blotting. A rat monoclonal anti-alpha-tubulin antibody (Cat. sc-53030 (YOL1/34), Santa Cruz Biotechnology, Dallas, Texas, USA) was used for western blotting. A rabbit polyclonal anti-αSMA antibody (Cat. Ab5694, Abcam) was used for western blotting. A rabbit polyclonal anti-GAPDH antibody (Cat. ab9485, Abcam) was used as a loading control for western blotting. A mouse monoclonal anti-myc antibody (Cat. 05–724, EMD Millipore, Burlington, MA, USA) was used for immunofluorescence and western blotting analyses. Antibodies against the N-terminus and C-terminus of STRA6 (Cat. PA5-43407 and Cat. PA5-59319, respectively; Invitrogen) were used for immunofluorescence and western blotting analyses. Goat polyclonal anti-human and anti-mouse albumin (Cat. AF3329-SP; R&D Systems, Minneapolis, MN, USA) was used for immunofluorescence analyses. Antibodies of HMGB1 (Cat. ab18256, Abcam) and HMGB1 (Cat. 3935S, Cell signaling) were used for immunofluorescence and western blotting analyses, respectively.

The siRNAs targeting human STRA6 (Cat. s34569) and human TIF1γ (Cat. 108771) were purchased from Thermo Fisher Scientific. The siRNA targeting mouse Stra6 was designed by Bioneer (Korea, Daejeon); the sequence is described above.

The RAR antagonist AGN193109 (Cat. 5758/10), the RXR antagonist PA452 (Cat. 5086/10), and the LXR blocker GSK2033 (Cat. 5694/10) were purchased from R&D Systems.

BODIPY (Cat. D3835, Thermo Fisher Scientific) was used to stain triglyceride in cells and tissues.

The pCMV6-STRA6 was purchased from ORIGENE. (Cat. RC227393).

### Primary HSC culture

The liver of a mouse was digested in situ as described previously [39]. The primary HSCs were isolated using the Nycodenz gradient method and incubated in DMEM supplemented with GlutaMAX (Gibco), 2% FBS, and 1% (v/w) penicillin/streptomycin (Cat. 15070063, Gibco). The cells were maintained at 37 °C in a humidified incubator with 5% CO_2_.

### Quantitative PCR analyses

RNA was isolated from cells using TRIzolTM reagent (Cat. 15596018) following the recommended protocol. RNA concentration was quantified using a NanoDrop spectrophotometer. Reverse transcription reactions contained 2 µg RNA and were performed using RT Master Premix(oligo d(T)) (Cat. EBT-1512; Elpisbiotech, Daejeon, Korea). Quantitative PCR was performed using FastStart Universal SYBR Green Master (Rox) (Cat. 4913850001; Merck, Darmstadt, Germany) and the Applied Biosystems^®^ 7500 system. The primer sequences were as follows: hPNPLA3 For, 5′-AGTCGTGGATGCCTTGGTATGT-3′; hPNPLA3 Rev, 5′-ATAGAAGGGGGACACGGTGA-3′l; hSTRA6 For, 5′-TCGCTGTCAATCCTTGTGCT-3′; hSTRA6 Rev, 5′-ATGAAAACAGCAGCAGGCAC-3′; hATGL(PNPLA2) For, 5′-CACCATCACAGTGTCCCCCT-3′; hATGL(PNPLA2) Rev, 5′-CATCTCTCGCAGCACCAGGG-3′; hSREBP1c For, 5′-CTGACCGACATCGAAGGTGA-3′; hSREBP1c Rev. 5′-AAGTGCAATCCATGGCTCCG-3′; hACL For, 5′-GACTTCGGCAGAGGTAGAGC-3′; hACL Rev, 5′-TCAGGAGTGACCCGAGCATA-3′; hACC For, 5′-TTAAGGGGTGAAGAGGGTGC-3′; hACC Rev, 5′-CCAAAAAGACCTAGCCCTCA-3′; hFASN For, 5′-CAGAGCAGCCATGGAGGAG-3′; hFASN Rev, 5′-TAGAGCCCCGCCTTCCAG-3; hSCD For, 5′-CTGCAGGACGATATCTCTAGCTC-3′; hSCD Rev, 5′-TCCAAGTAGAGGGGCATCGT-3′; hELOVL6 For, 5′-CAGGAGAACACTCGAAATCAAGC-3′; hELOVL6 Rev, 5′-TTTCTTCCAGTTTTCCTGCATCC-3′; mStra6 For, 5′-CCAGTCACATCCAGGAGTCATA-3′; mSttra6 Rev, 5′-GCATCCCTTCTTCTTCTTCTG-3′; mSrebp1c For, 5′-CAAAAGCAAATCACTGAAGGACC-3′; m Srebp1c Rev. 5′-CGGGCTCAGAGTCACTACCAC-3′; mACL For, 5′-GAAGCACCCAGAAGGCAAGAT-3′; mACL Rev, 5′-CGGACAAAGATGGTGACCTCA-3′; mAcc For, 5′-GCTGAGATTGAGGTAATGAAGATGG-3′; mAcc Rev, 5′-AGCCTGTTGAACTTTACTGGGG-3′; mFasn For, 5′-TGGAAGGCTGGGCTCTATG-3′; mFasn Rev, 5′-CGGAGTGAGGCTGGGTTG-3; mScd For, 5′-CCTCTTCGGGATTTTCTACTACAT-3′; mScd Rev, 5′-TGGCAATGATAAGGAAGATCCG-3′; mElov16 For, 5′-GCAGGAAAACTGGAAGAAGTCT-3′; mElov16 Rev, 5′-GAAGAGCACCGAATATACTGAAGA-3’.

### Immunofluorescence analyses

Cells were fixed with 4% paraformaldehyde for 15 min and blocked with 5% normal horse serum for 30 min. Where required, permeabilization was performed by incubating with 0.5% Triton X-100 in 1 × PBS for 5 min. Liver tissues were fixed with 4% paraformaldehyde for 7 days, and the fixatives were changed every 2 days. Sucrose was exchanged to a final concentration of 30% to liver tissue, and a tissue block was made using OCT compound at temperatures below – 20 °C. For immunofluorescence, 7 µm thick sections of liver tissues were mounted onto glass slides. The slides washed out with PBS enough, next process was same as cell’s process. Retinol was detected using a 355 or 405 nm laser. Images were acquired using an LSM710 (Carl Zeiss, Göttingen, Germany) or Leica confocal microscope.

### Western blotting

Whole cell lysates and supernatants of cells were collected and lysed with RIPA lysis and extraction buffer (Cat. 89900, Thermo Fisher Scientific). Proteins were quantified using the BCA method (Cat. 23223, Thermo Fisher Scientific), and approximately 40–60 µg protein was loaded onto an SDS-PAGE gel for analysis. The primary antibodies are described above.

### Oil-red O

Oil red-O (0.5 g) was dissolved in 100 mL isopropanol in a warm water bath. This stock solution was diluted 3:2 in clean water and filtered to remove insoluble aggregates. Frozen tissue Sections (8–10 µm thick) were air-dried and then rinsed under running water for 10 min. The buffer was changed to 60% isopropanol, and the slides were submerged in oil red-O stain solution for 15 min and counterstained with hematoxylin. The slides were then rinsed with clean water and mounted in aqueous mounting solution.

### Picro-sirius staining

A 0.5 g sample of Direct Red 80 (Cat. 365548, Sigma-Aldrich) was mixed in a saturated aqueous solution of 500 mL picric acid. Acidified water was prepared by mixing 5 mL acetic acid with 1 L clean water. The samples were dewaxed and counterstained with hematoxylin for 8 min. Subsequently, the slides were washed in running water for 10 min and stained with picro-sirius red for 1 h. Next, the slides were washed twice with acidified water and then dehydrated by washing three times with 100% EtOH. Finally, the samples were cleaned with xylene and mounted in organic mounting solution.

### Flow cytometry

Retinol autofluorescence was detected at 450–500 nm using a BD FACS Canto™II analyzer (Becton Dickinson, Franklin Lakes, New Jersey, USA). Hoecst33258 staining was used as a positive control for the retinol wavelength.

### Luciferase assay

LX2 cells were transfected with the pGL3-RARE construct (Cat. 13458; Addgene, Waterown, MA, USA) and the pRL-renilla construct (Cat. E2231; Promega, Madison, WI, USA) as a positive control for transfection efficiency. The Dual-Luciferase Reporter Assay System (E1910, Promega) and the GloMax^®^ Plate Reader (Promega) were used to detect luciferase activity.

### ALT/AST assay

Analyses were performed using the Aspartate Aminotransferase Activity Assay Kit (Cat. ab138878, Abcam) and the Alanine Transaminase Activity Assay Kit (Cat. ab105134, Abcam). Blood serum and standard curve prepared and reaction mixture added on each sample 100 µl. The activities of ALT and AST were measured at OD570 and OD450 nm, respectively, at 70 min intervals. The activities were determined as recommended by the manufacturer’s protocols.

## Statistical analysis

Statistical analysis was performed using the GraphPad Prism 6 software (GraphPad Software, La Jolla, CA, USA). Data expressed as the mean ± standard deviation. Differences between groups were analyzed by the unpaired t-test or one-way analysis of variance (1-way ANOVA). P-values < 0.05 were considered statistically significant.

## Supplementary Information

**Additional file 1: Figure S1.** A. Immunofluorescent detection of HMGB1 (green) and nucleus (DAPI, blue) in the liver tissue of normal and Lrat:Cas9-ERT2: sgTif1γ mice treated with TMX. The DIC image shows the phenotype of the cells. Scale bar: 50 μm. B. Western blotting of supernatant of HepG2 cells treated retinol for 3 days or 7 days. Western blotting was performed using an anti-HMGB1 antibody and a Ponceau staining was used for loading control. **Figure S2.** Immunofluorescent detection of albumin (magenta) and BODIPY staining of triglyceride (red) in HepaRG cells treated with or without retinol. The nuclei were stained with SYTOX green. The DIC image shows the phenotype of the cells. Scale bar: 5 μm. **Figure S3.** A. Retinol autofluorescence (blue) in LX2 cells treated with or without retinol. The nuclei are shown in green. Scale bar: 25 μm. B. Flow cytometry analyses of the cells described in A. C. The expression levels of the mRNAs encoding human ATGL, PNPLA3, and STRA6 in LX2 cells treated with or without recombinant human TGFβ1 and/or recombinant human HGF. D. The expression level of the mRNA encoding human STRA6 in LX2 cells overexpressing TIF1γ or transfected with a TIF1γ-specific siRNA (100 nM) for 2 days. For overexpression, the cells were transfected with pLenti-TIF1γ and incubated for 1 day prior to analysis. **Figure S4.** Myc-tagged human STRA6 was transfected into 293 T cells, and the cells were treated with or without 1 μM retinol for 1 day. Western blotting was performed using an anti-STRA6 antibody and an anti-myc antibody. GAPDH was detected as a loading control.

## Data Availability

Not applicable.

## Abbreviations

ACC: Acetyl CoA carboxylase
ACL: ATP citrate lyase
ATR: All-trans-retinol
ELOVL6: ELOVL fatty acids elongase 6
FASN: Fatty acid synthase
HMGB1: High mobility group box 1
HSC: Hepatic stellate cell
LXR: Liver X receptor
RAR: Retinoic acid receptor
RXR: Retinoid X receptor
SCD: Stearoyl-CoA desaturase-1
SREBP1: Sterol regulatory element-binding transcription factor 1
STRA6: Stimulated by retinoic acid 6
TAA: Thioacetamide
TIF1γ: Transcriptional intermediary factor 1γ
TMX: Tamoxifen
