# Supplementary material for: Retinol from hepatic stellate cells via STRA6 induces lipogenesis on hepatocytes during fibrosis
Source: Cell Biosci. 2021 Jan 6;11:3. doi: 10.1186/s13578-020-00509-w (PMC7789180; doi:10.1186/s13578-020-00509-w)
Supplement: Supplementary file 1 — Additional file 1: Figure S1. A. Immunofluorescent detection of HMGB1 (green) and nucleus (DAPI, blue) in the liver tissue of normal and Lrat:Cas9-ERT2: sgTif1γ mice treated with TMX. The DIC image shows the phenotype of the cells. Scale bar: 50 μm. B. Western blotting of supernatant of HepG2 cells treated retinol for 3 days or 7 days. Western blotting was performed using an anti-HMGB1 antibody and a Ponceau staining was used for loading control. Figure S2. Immunofluorescent detection of albumin (magenta) and BODIPY staining of triglyceride (red) in HepaRG cells treated with or without retinol. The nuclei were stained with SYTOX green. The DIC image shows the phenotype of the cells. Scale bar: 5 μm. Figure S3. A. Retinol autofluorescence (blue) in LX2 cells treated with or without retinol. The nuclei are shown in green. Scale bar: 25 μm. B. Flow cytometry analyses of the cells described in A. C. The expression levels of the mRNAs encoding human ATGL, PNPLA3, and STRA6 in LX2 cells treated with or without recombinant human TGFβ1 and/or recombinant human HGF. D. The expression level of the mRNA encoding human STRA6 in LX2 cells overexpressing TIF1γ or transfected with a TIF1γ-specific siRNA (100 nM) for 2 days. For overexpression, the cells were transfected with pLenti-TIF1γ and incubated for 1 day prior to analysis. Figure S4. Myc-tagged human STRA6 was transfected into 293 T cells, and the cells were treated with or without 1 μM retinol for 1 day. Western blotting was performed using an anti-STRA6 antibody and an anti-myc antibody. GAPDH was detected as a loading control. [file 13578_2020_509_MOESM1_ESM.pdf]

Figure S1

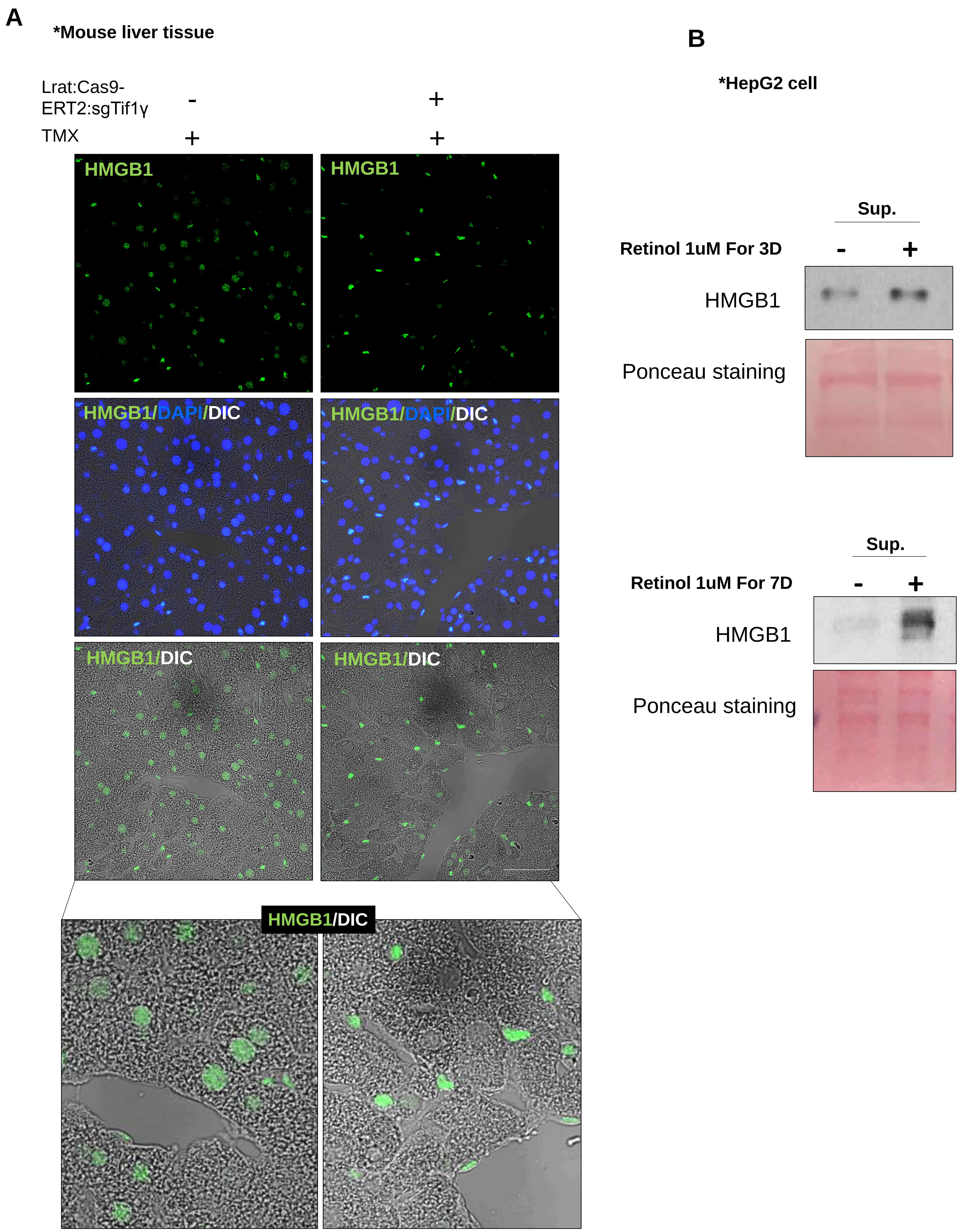
A
*Mouse liver tissue
Lrat:Cas9-ERT2:sgTif1γ
-
+
TMX
+
+
HMGB1
HMGB1
HMGB1/DAPI/DIC
HMGB1/DAPI/DIC
HMGB1/DIC
HMGB1/DIC
HMGB1/DIC
B
*HepG2 cell
Sup.
Retinol 1uM For 3D
-
+
HMGB1
Ponceau staining
Sup.
Retinol 1uM For 7D
-
+
HMGB1
Ponceau staining

Figure S2

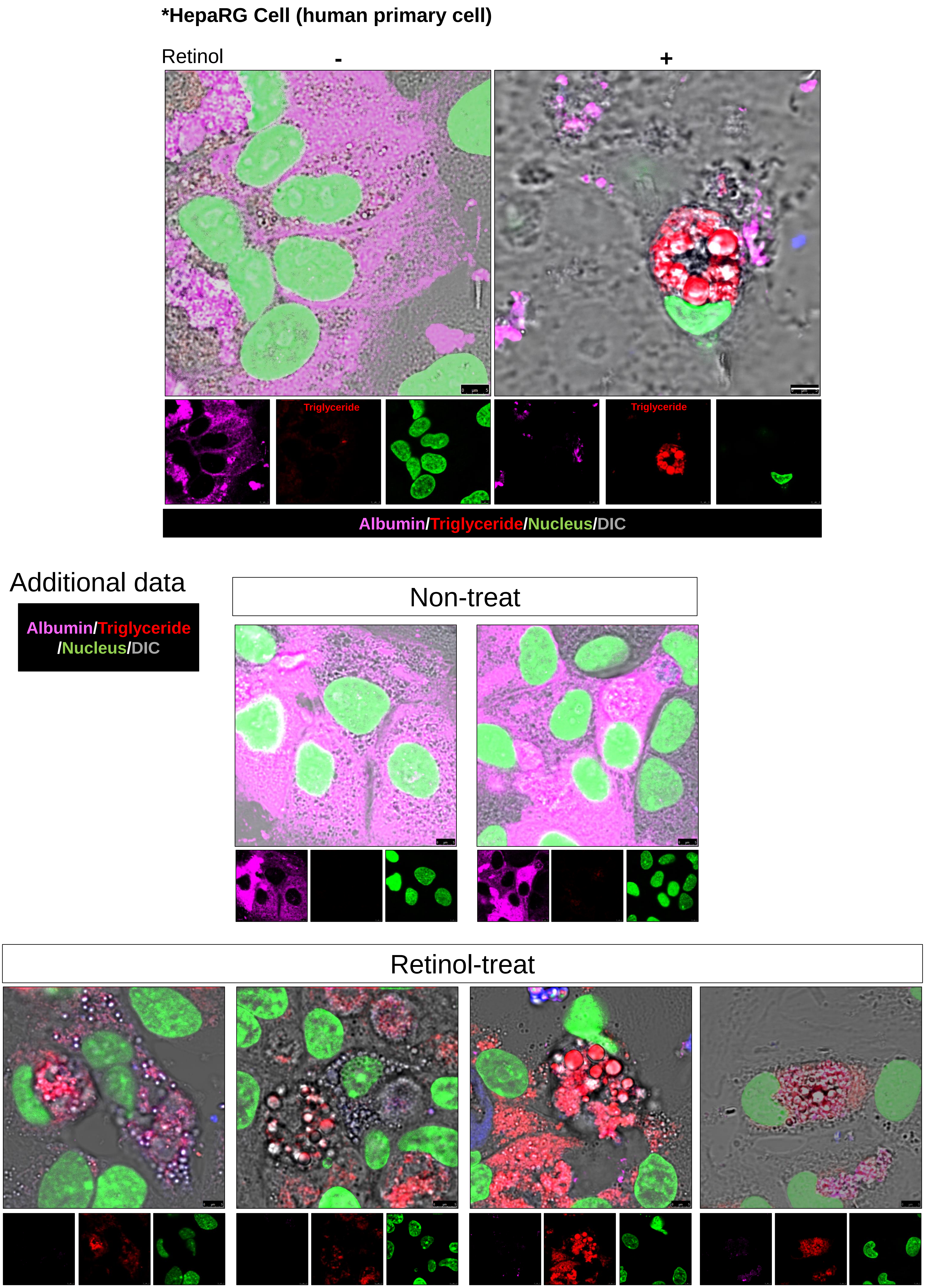
*HepaRG Cell (human primary cell)
Retinol
-
+
Triglyceride
Triglyceride
Albumin/Triglyceride/Nucleus/DIC
Additional data
Albumin/Triglyceride
/Nucleus/DIC
Non-treat
Retinol-treat

Figure S3

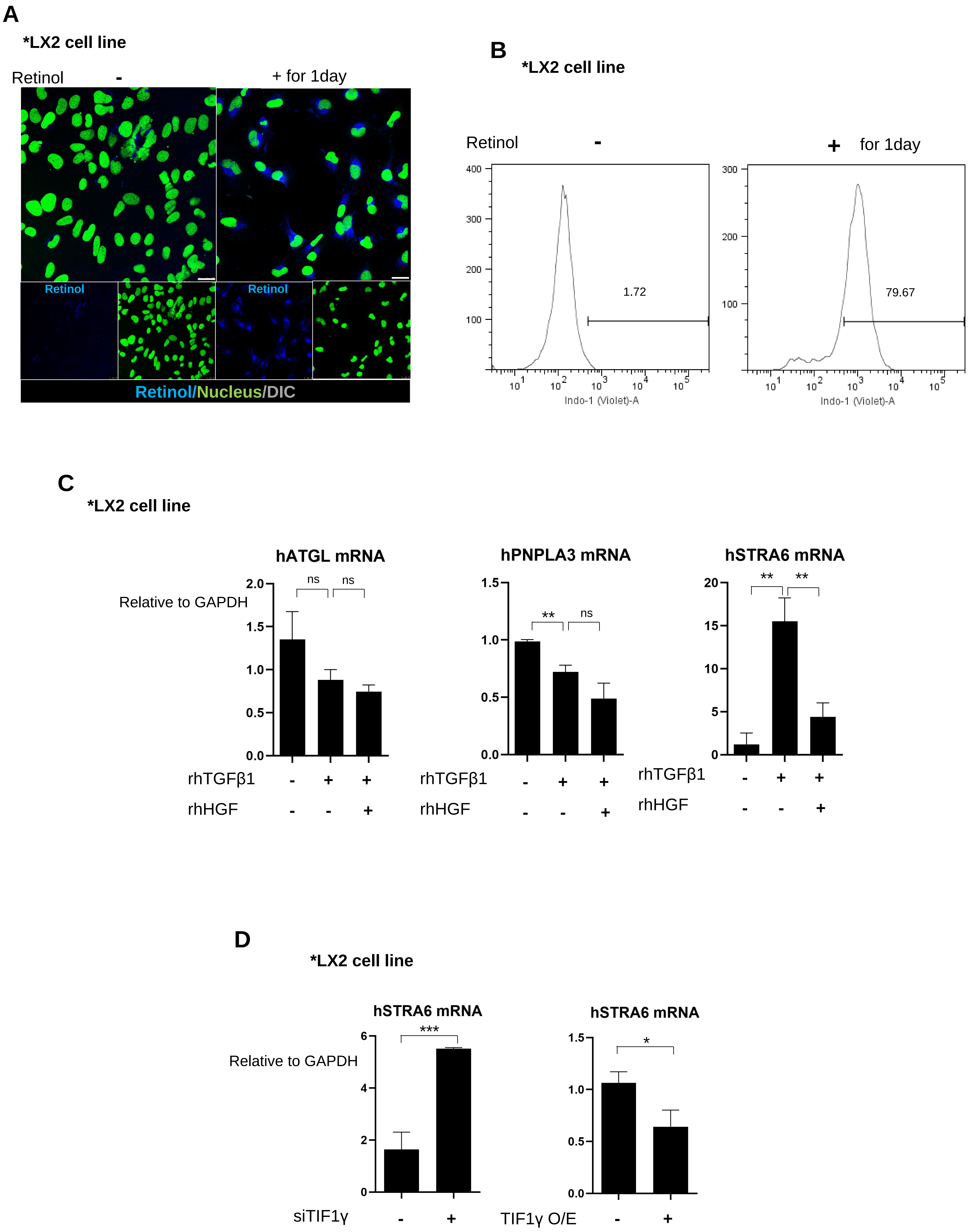

A
*LX2 cell line
Retinol -
+ for 1day
Retinol
Retinol
Retinol/Nucleus/DIC
B
*LX2 cell line
Retinol -
+ for 1day
1.72
79.67
Indo-1 (Violet)-A
Indo-1 (Violet)-A
C
*LX2 cell line
hATGL mRNA
hPNPLA3 mRNA
hSTRA6 mRNA
Relative to GAPDH
ns
ns
**
ns
**
**
rhTGFβ1 - + +
rhHGF - - +
D
*LX2 cell line
hSTRA6 mRNA
hSTRA6 mRNA
Relative to GAPDH
***
*
siTIF1γ - +
TIF1γ O/E - +

Figure S4

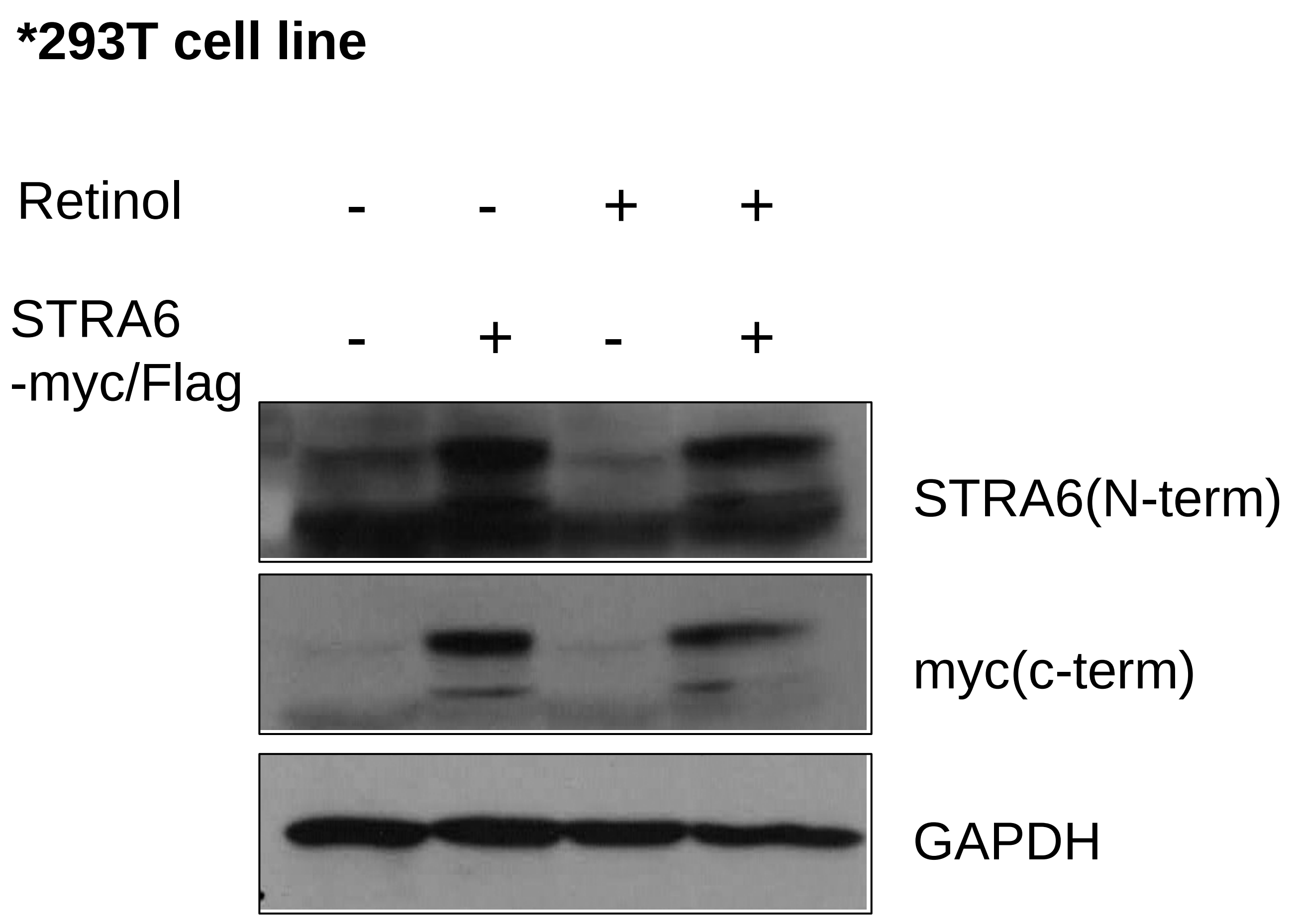
*293T cell line
Retinol - - + +
STRA6
-myc/Flag - + - +
STRA6(N-term)
myc(c-term)
GAPDH
